# Development and implementation of a gyrolab-based generic anti-drug antibody assay for antibody-drug conjugates in cynomolgus monkey studies

**DOI:** 10.3389/fimmu.2025.1711816

**Published:** 2025-11-18

**Authors:** Runzhong Fu, Christine O’Day, Razieh Esmaeili, Stavros Zinonos, Arlan Martin, Lisa McCulloch, Charles Y. Tan

**Affiliations:** 1 ADC Bioanalytical Group, Pharmacokinetics Dynamics and Metabolism Department, Pfizer Inc., Bothell, WA, United States; 2 Immunogenicity Sciences Group, Pharmacokinetics Dynamics and Metabolism Department, Pfizer, Andover, MA, United States; 3 ECD Group, Clinical and Research Assay Statistics, Pfizer Inc., Pear River, NY, United States

**Keywords:** anti-drug antibodies, immunogenicity, non-clinical, antibody drug conjugates, bioanalytical assays, gyrolab

## Abstract

**Introduction:**

Non-clinical immunogenicity yields valuable insights into pharmacokinetics, efficacy, and safety. Given the complex nature of ADC therapeutics, early detection of anti-drug antibodies (ADAs) is critical for elucidating exposure and toxicity issues that may be translated into the clinic. A universal ADA assay employing generic reagents and a standardized cut point in nonclinical studies can enhance cost efficiency, expedite development, and address limitations indrug tolerance associated with traditional bridging assays.

**Methods:**

Gyrolab-based generic ADA assay was carried out by initially spiking ADC into cynomolgus samples at a concentration of 300 mg/mL. Drug-ADA complexes were isolated using an anti-human Fc antibody, followed by detection with an anti-cynomolgus detection antibody. A total of 22 distinct ADCs were assessed across 50 cynomolgus subjects, each evaluated in six replicates to determine the cut point. Sensitivity and positive control (PC) assessments were performed by titrating an anti-human light chain generic surrogate positive control.

**Results and Discussion:**

A Gyrolab-based ADA assay with a universal cut point of 2.64 was developed, with 16 out of 22 ADCs acceptable for producing a standardized cut point. The average sensitivity of the positive control was 99.5 ng/ml and drug tolerant up to 1 mg/mL. Factors such as various linkers, drug antibody ratio (DAR), or backbone variations (variable region, Fc mutants and engineered cysteines) had minimal effect; however, one antibody variable region gave elevated signals. A workflow for assay used in Good Laboratory Practice (GLP) study was established, and its application demonstrated in a case study.

## 1 Introduction

Antibody-based therapeutics can elicit unwanted immune response known as immunogenicity (1–4). The hall mark of immunogenicity is production of anti-drug antibodies (ADA). ADAs can bind to the antibody therapeutic and affect the efficacy (1, 5), pharmacokinetics (PK) (1) and pharmacodynamics (PD), and immune related safety profile (6, 7) in the clinic. For this reason, ADA testing has become routine in both preclinical and clinical assessments (4, 8–11).

A typical ADA assessment in the clinic follows a standard three-tiered approach, consistent of a screening assay, confirmation assay and a characterization assay (12, 13). The screening assay assesses the presence of antibodies that binds to the therapeutic, which is followed by a confirmation assay confirming the specificity of ADA towards biotherapeutic by direct competition. Characterization assays mainly include a titration assay that measures the magnitude of ADA response and a neutralizing antibody (NAb) assay that assesses the ability of ADA to interfere with therapeutic effect (12, 13). Additional characterizations such as isotyping, epitope mapping and cross-reactivity may also be carried out based on immunogenicity risks (11, 12). In recent years, health governance agencies have recommended a risk-based approach, evaluating both intrinsic and extrinsic factors of the biotherapeutic to provide tailored strategy for clinical ADA monitoring (11, 14). Incidence of treatment-induced ADA, ADA titer, persistently positive ADA and NAb incidence are key factors in interpretating clinical immunogenicity and explaining clinical outcomes (5, 14).

Preclinical ADA is not predictive of immunogenicity in the clinic (15). However, monitoring ADA in preclinical studies can still be critical to understand *in vivo* profiles of the monoclonal antibody (mAb) therapeutics, especially when there is unexpected change in PK, PD and tox endpoints (15, 16). Due to different objectives of preclinical ADA monitoring, preclinical ADA assays are less stringent than clinical assays and have historically applied a “lean” approach of two tiers (screening and confirmatory or titer) (17). In recent years, industrial opinions towards preclinical ADA assay further shifted to a more simplified approach of to only applying a screening assay with 0.1-1% false positive rate (FPR). A leaner validation strategy with only screening cut point (SCP), sensitivity, drug tolerance and precision as required parameters were recommended. When semi-quantification of ADA response magnitude is required, using signal-to-noise (S/N) ratios from the screening assay is considered as an alternative to titer (18, 19).

The detection of both clinical and preclinical ADA typically utilizes a bridging assay with both labelled mAb therapeutic as both capture and detection reagent (12). Although being a gold-standard ADA assay format, the assay performance is oftentimes challenged by circulating drug and target. Under excessive drug condition, ADA can be present as free-form, drug-ADA complex and/or sometimes drug-target-ADA complex form (20). To capture total ADA sample pretreatments dissociating the ADA from therapeutic drug are usually required (21–25). However, sample manipulation process could lead to poor ADA recovery and worsened target interference. Given the high drug dose used in preclinical toxicokinetic (TK) studies compared to clinical studies, high drug tolerance is essential for ADA assay performance.

Antibody-drug conjugates (ADCs) are oncology therapeutics consisting of monoclonal antibodies linked to small molecule payloads, typically cytotoxic agents such as auristatins. ADCs are diverse in conjugation chemistry, linkers and payloads (26). ADCs can also trigger treatment-induced ADA in the clinic with rates ranging from 0~36% (27). Majority of ADA responses in the clinic are directed at the antibody component rather than the linker or payload. With recent development of ADCs incorporating immune-stimulating payloads, such as pattern recognition receptor (PRR) agonists, there is an increased risk of generating ADA specific to the payload or linker (28). In preclinical studies, ADAs have been reported to target both linker and payload as well as antibody backbone (29, 30). Early ADA profiling of ADCs in preclinical species, including domain mapping, can help better interpret immunotoxicity and PK/PD outcomes. The standard bioanalytical method for monitoring preclinical ADA in ADCs is comparable to that used for mAb therapeutics and utilizes a specific bridging assay with sample pretreatment. However, ADCs may face more challenges with a traditional bridging assay. Labelling and assay buffer selection for ADCs is more intricate because of the potential for changes in biophysical properties caused by hydrophobic payloads and steric effects (30, 31).

Generic ADA methods have been used to characterize ADA in nonclinical studies for mAb therapeutics. Samples were first treated with excess drug to form a drug-ADA complex, which was further pulled down by an anti-human constant region antibody and detected by a nonclinical specie-specific detection antibody (32). This assay format detects total ADA and addresses drug interference issues found in standard bridging assays. This novel approach of detecting ADA for mAb therapeutics have been explored on ELISA (33, 34), electrochemiluminescence (ECL) (35) and Gyrolab platforms (36). However, limited studies reported on using the generic ADA approach to assess immunogenicity for ADCs. The previously reported ELISA-based generic ADA showed good drug tolerance and sensitivity for ADCs, but had a limited dynamic range, typical of colorimetric ELISA (37). This method may present limitations for wide application in light of the ongoing transition to using S/N instead of titer. Gyrolab is automated immunoassay platform that utilizes a microfluidic compact disc (CD) (38). When comparing platforms for both PK and ADA performance, Gyrolab demonstrates an expanded dynamic range, enhanced sensitivity, superior signal-to-noise ratio, and greater drug tolerance relative to ELISA (39, 40). Recent reports have also shown that Gyrolab is able to improve the dynamic range of generic ADA assays for mAb therapeutics (36). Another notable gap is that many generic ADA papers did not specify a universal cut point or outline a workflow for assessing the suitability of new test articles.

In this study, we present a generic ADA assay on the Gyrolab platform, with an emphasis on ADC therapeutics. A total of 22 ADCs were evaluated, representing diverse complementarity determining regions (CDRs), backbone mutations, linker technologies, and payloads. We successfully established a universal cut point factor applicable to a subset of these ADCs and defined criteria for inclusion or exclusion when assessing new test articles. Additionally, we investigated principal component contributors within ADCs that influenced the results. An application of this assay in evaluating the domain specificity of ADA towards ADC was also demonstrated.

## 2 Methods

### 2.1 Materials

Rexxip™ HX (cat #P0020033), F buffer(cat #P0004825), Gyros wash buffer duo solution (cat #P0004825), and Gyrolab Bioaffy™ 1000 CD (cat #P0004253) were from Gyros Protein Technologies (Uppsala, Sweden). Gyros wash solution PBS-T (0.01%) were prepared with 10x PBS (cat #70011044) from Gibco (New York, USA) and Tween-20 (cat #P2287) from Sigma-Aldrich (Missouri, USA). PH 11 buffer packs (cat #P0020096) were from Gyros Protein Technologies (Uppsala, Sweden). Assay buffer (5% BSA, 0.1% Tween20, 0.3M NaCl solution, 0.1M Tris, pH8.5) solutions were prepared in-house with bovine serum albumin (cat # A7030) and tween-20 (cat #P1379) from Sigma-Aldrich (Missouri, USA), Tris, 0.5M buffer solution pH 8.5 (cat #J622131.AP) and sodium chloride solution (5M) (cat #AM9759) from Thermo Fisher Scientific (Massachusetts, USA). Individual cyno samples were obtained from BioIVT (New York, USA) and pooled in-house. AcroPrep™ Advance 96-well 350 µL, 0.2 µm supor membrane filter plates (cat #8019) were obtained from Cytiva life sciences (Marlborough, MA).

### 2.2 Preparation of critical reagent antibodies

The capture antibody was CaptureSelect™ Human IgG-Fc PK Biotin Conjugate antibody (cat #7103322100) from Thermo Fisher Scientific (Massachusetts, USA). The detection antibody used was a mouse anti-monkey IgG that does not cross-react with human IgG (cat #SA1-10653), sourced from Thermo Fisher Scientific (Massachusetts, USA) and labelled in-house. The unlabelled antibody was buffer exchanged into 1x PBS, pH 7.4 (cat #10010-031) using Slide-A-Lyzer™ Dialysis Cassettes, 10K MWCO (cat #66381, Thermo Fisher Scientific, Massachusetts, USA). The buffer-exchanged monoclonal antibody was subsequently labeled with Alexa Fluor 647 NHS Ester (Succinimidyl Ester) labelling kit (cat #A20006, Thermo Fisher Scientific, Massachusetts, USA) in accordance with the manufacturer’s instructions. Performance was validated using a functional assay.

### 2.3 Generic positive control

Surrogate generic positive control (PC) antibody was an anti-human kappa light chain (LC) IgG mouse/cyno chimeric antibody. The mouse anti-human kappa LC IgG was developed at Abveris, Inc. (Massachusetts, USA) by immunizing mice with human FAb. After rapid immunization, B-cells from mice with adequate human mAb titer were isolated and screened using the Berkeley Lights Beacon Optofluidic system. B-cells with the desired response were isolated, and variable heavy and light chain sequences were identified using NGS. Sequences were cloned into a proprietary expression vector, expressed as mouse mAbs in HEK293 cells, and purified. Specificity and binding kinetics were analyzed using indirect ELISA and BLI. A reformatted mouse antibody was created by replacing its IgG2a heavy chain and Kappa light chain constant domains with cynomolgus macaque IgG1 and Lambda domains, respectively, then expressed in HEK293 and purified by ATUM to produce a mouse/cyno chimeric mAb. The functionality of the reformatted chimeric mAb was assessed using BLI characterization.

### 2.4 gADA assay

Samples and quality controls (QC) were diluted at a ratio of 1:20 in Rexxip-HX buffer, thoroughly mixed, and transferred to AcroPrep Advance 96-well filter plates. A receiving plate was placed underneath, and the assembly was centrifuged at 3900 rpm for 4 minutes. A complexation buffer was made by preparing 3750 ng/mL of the test article in investigation in assay buffer, for a final concentration of 3000 ng/mL test article post MRD. The filtered solution was further diluted 1:5 into the complexation buffer and incubated at room temperature with shaking for 1–2 hrs. The capture antibody was diluted in assay buffer to 100 µg/mL, and detection antibody was prepared in Rexxip F buffer at 50 nM. After designing the 1000-3W-019-X 4 method in Gyrolab manager software, load the samples, QC, capture and detection reagent, and wash buffer duo onto a 96 well PCR plate according to loading list plate map. Cover with foil plate sealer and load into the instrument. Load the wash station with filtered PBST and PH11 buffer. Prime and start the Gyrolab run through Gyrolab client software. Response is read by the Gyrolab instrument at 1% Photomultiplier tube (PMT) setting. Data analysis was carried out automatically through Gyrolab evaluator software. Assay conditions including minimum required dilution (MRD) buffer, complexation buffer, wash buffer and gyrolab CDs were screened during development to minimize matrix background. Key development data and methods are included in **Supplementary Figure 1**.

### 2.5 Cut point and PC screening

Cut point screenings were carried out for each ADC test article by running 50 naïve cyno (25 males and 25 female) individual plasma with the gADA method mentioned above. A pooled sample was included on each CD. Each sample repeats were analyzed as duplicates on Gyrolab CD. Six repeated screenings were carried out on different days with three different Gyros Xpand instruments between two analysts. PCs were prepared with the same 50 individual and pool plasmas, with LPC set as 650 ng/mL, MPC as 40,000 ng/mL and HPC as 100,000 ng/mL. The signal-to-noise was calculated by normalizing to the average pool on each CD for both naïve individuals (NC) samples and PC samples and used for floating cut point calculation.

### 2.6 Drug tolerance

Drug tolerance was tested in four ADCs with different levels of estimated cut points to determine the amount of drug for complexation. Briefly, 1.33x PC was prepared in cyno pooled plasma and 4x ADC drug concentrations was prepared in PBST. 15 µL 1.33x PC and 5 µL 4x ADC drug were mixed and to have final concentrations of 1x PC levels at 100, 200, 650, 1000 and 2000 ng/mL and 1x drug concentration at 98, 480, 2400, 12000, 60000, 300000, 1000000 ng/mL. The TA/ADC mixed solution were further diluted 20-fold in Rexxip-HX buffer and then diluted 5-fold in assay buffer for a final MRD of 1:100. After 1 hr incubation at room temperature, samples were loaded on a 96-well PCR plate and ran with gADA assay protocol. The amount of drug spikes was determined by the level of drug tolerance divided by 100 to adjust for MRD in the final incubation solution.

### 2.7 Sensitivity assessment

Four independent sensitivity curves were generated by two analysts and evaluated over a four-day period. PC was serial diluted in pooled cyno plasma to 38, 57.1, 85.6, 128.4, 192.6, 288.9, 433.3, 650, 975, 1462.5, 2193.8 ng/mL. The curve samples were diluted 1:20 in Rexxip HX, which was further diluted 1:5 in complexation buffer containing the specific ADC under investigation. The mixture was incubated at rt for 1hr and loaded onto the Gyrolab instrument. Capture, detection and wash buffer reagents were prepared as the gADA protocol. Similarly, 1000-3W-019-X 4 method was run with 1% PMT response acquired. Signal-to noise data was calculated by normalizing the curve response for blank matrix average response on each CD. Dose response curves were modelled using the 4-PL method in R 4.5.1.

### 2.8 Instrument and software

Generic ADA response (relative fluorescence units) was measured on a Gyrolab^®^ xPand with 1%PMT using Gyrolab^®^ software v8.1. ELISA was read on Clariostar plate reader with software BMG Labtech 4.01R2. The raw data generated was processed using R software 4.5.1, Microsoft Excel and Graphpad Prism10. All graphics were created in Biorender.

### 2.9 Statistics

Outliers samples for each of the ADC test articles were identified by first log transforming the S/N data normalized to naïve cyno pool on each CD and evaluating the upper and lower limit of 1.5 times the interquartile range (IQR) calculation. Log-transformed data outside the set bounds are excluded as outliers. Before cut point analysis, plasma reactivity in naïve individuals was screened, excluding outliers (see **Supplementary Figure 3**). A single cut point at the 95th percentile was identified for a combined group of 16 similar studies using quantile regression, with ADC as the independent variable and S/N value as the dependent variable. Consistency of the 95^th^ percentile among the combined 16 studies was assessed using a chi-squared test of homogeneity (chi-squared test, **Table 1**); other studies with different tails were excluded.

**Table 1:** Estimated cut point for each of the ADCs.

TA	IgG subclass	Cut point	Cut point excluding outliers	Cut point-all naive samples
mAb5-Linker2 (4)	IgG1	2.08	14/270 = 5.2%	42/298 = 14.1%
h00-Linker1 (4)	IgG1	2.84	14/270 = 5.2%	44/300 = 14.7%
mAb2-Linker3 (8)	IgG1	2.61	14/270 = 5.2%	44/300 = 14.7%
*mAb2-Linker1 (4)	IgG1	3.61	14/270 = 5.2%	44/300 = 14.7%
*mAb1-Linker3 (8)	IgG1	6.79	15/294 = 5.1%	21/300 = 7.0%
*mAb4-Linker6 (4)	IgG1	2.33	15/294 = 5.1%	21/300 = 7.0%
mAb5-Linker1 (4)	IgG1	2.34	14/270 = 5.2%	44/300 = 14.7%
*mAb3/mut7-Linker5 (4)	IgG1	3.71	14/276 = 5.1%	38/300 = 12.7%
*h2A2-Linker6 (4)	IgG1	6.05	15/294 = 5.1%	21/300 = 7.0%
h00-Linker7 (4)	IgG1	2.83	14/270 = 5.2%	44/300 = 14.7%
h00-Linker6 (4)	IgG1	2.24	14/270 = 5.2%	44/300 = 14.7%
h00-Linker3 (8)	IgG1	2.70	14/270 = 5.2%	44/300 = 14.7%
h00 ec2/mut2-Linker4 (2)	IgG1	3.26	14/270 = 5.2%	43/300 = 14.3%
h00/mut6-Linker3 (4)	IgG1	2.54	14/270 = 5.2%	44/300 = 14.7%
h00/mut5-Linker1 (4)	IgG1	2.83	14/270 = 5.2%	43/300 = 14.3%
h00 ec2/mut3-Linker1 (2)	IgG1	2.38	14/270 = 5.2%	43/300 = 14.3%
h00/mut8-Linker1 (4)	IgG1	2.40	14/270 = 5.2%	44/300 = 14.7%
h00/mut7-Linker1 (4)	IgG1	2.49	14/270 = 5.2%	44/300 = 14.7%
*h00 ec4/mut1-Linker1 (4)	IgG1	1.88	14/276 = 5.1%	38/300 = 12.7%
mAb4/mut9-Linker1 (4)	IgG1	2.13	14/270 = 5.2%	44/300 = 14.7%
h00 ec4/mut4-Linker1 (4)	IgG1	2.94	14/270 = 5.2%	44/300 = 14.7%
h00-Linker4 (8)	IgG1	3.07	14/270 = 5.2%	44/300 = 14.7%

*ADCs are excluded from universal cut point analysis due to failed homogeneity test.

Canonical correlation analysis (CCA) was performed to identify the primary contributing components within the ADC. The optimal linear combination of ADC components selected to maximize the correlation between ADC-specific cut points and components demonstrated a correlation coefficient of 0.98 (Rao’s F = 0.0003) with study-specific cut points. Interpretation of the CCA results relied on standardized loadings and boxplots of the first canonical variable, categorized accordingly.

Additionally, a two-sided binomial test was applied to determine acceptance regions for the 85th percentile in a new study with sample size 30, establishing that a value of 25 is consistent with the 85th percentile across the combined 16 studies (41).

## 3 Results

### 3.1 A generic ADA method provides a simplified, drug-tolerant alternative for TK assessment in cynomolgus studies.

**Figure 1:**
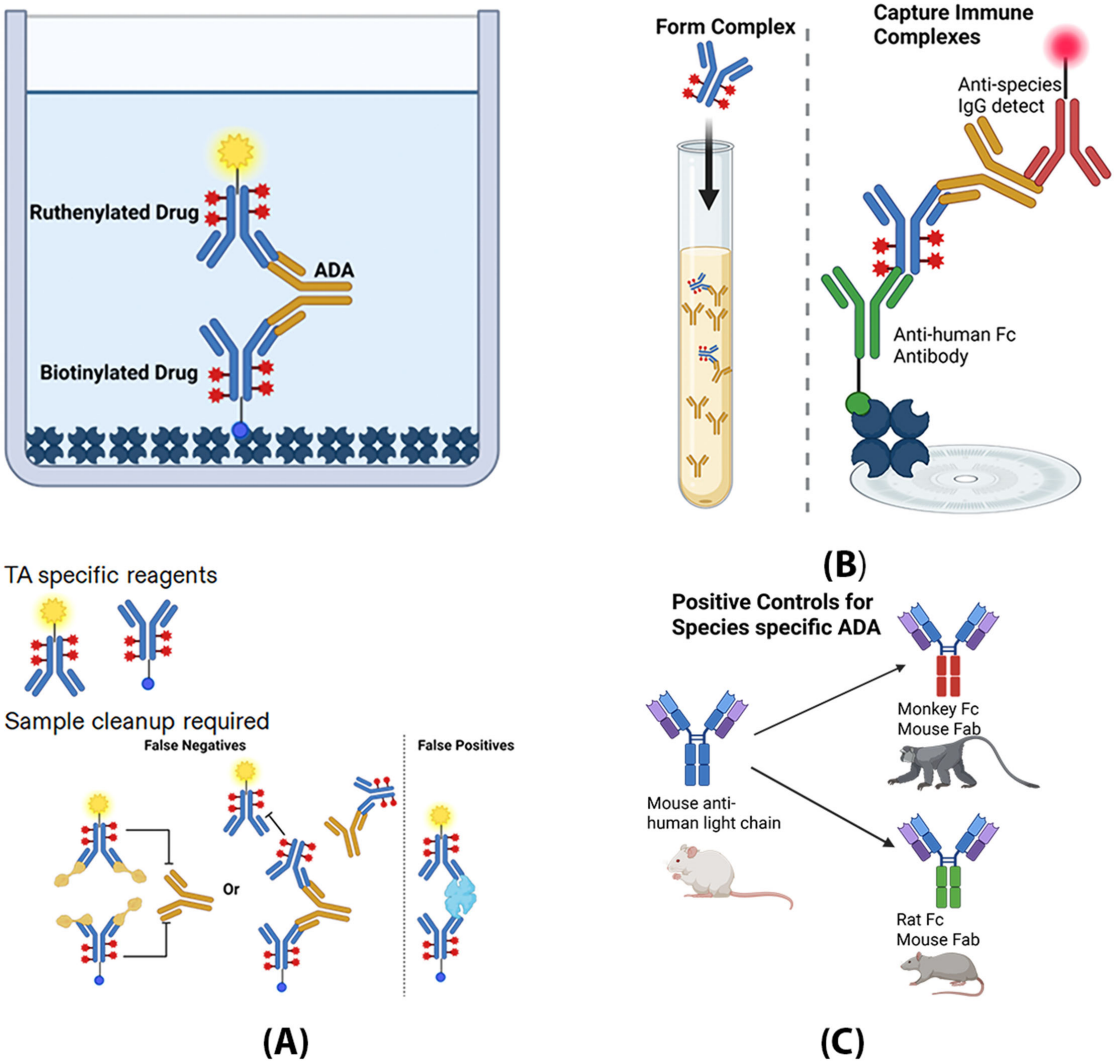
Generic and specific ADA assay formats. Panel **(A)** displays an ADA bridging assay that uses labelled drug as both capture and detection reagents. This approach requires dedicated reagents for each program and involves acid dissociation to process the sample. **(B)** The gyrolab-based generic ADA assay introduces drug to bind all free ADA; immunocomplexes are then collected using an anti-human Fc antibody and detected with an anti-species reagent. **(C)** A chimeric anti-human light chain antibody functions as a generic surrogate positive control for assay development and evaluation. This reagent is created by producing a mouse antibody and substituting the heavy chain portion with those from specific nonclinical species, such as cynomolgus monkey or rat, tailored for each ADA assay.

**Figure 2:**
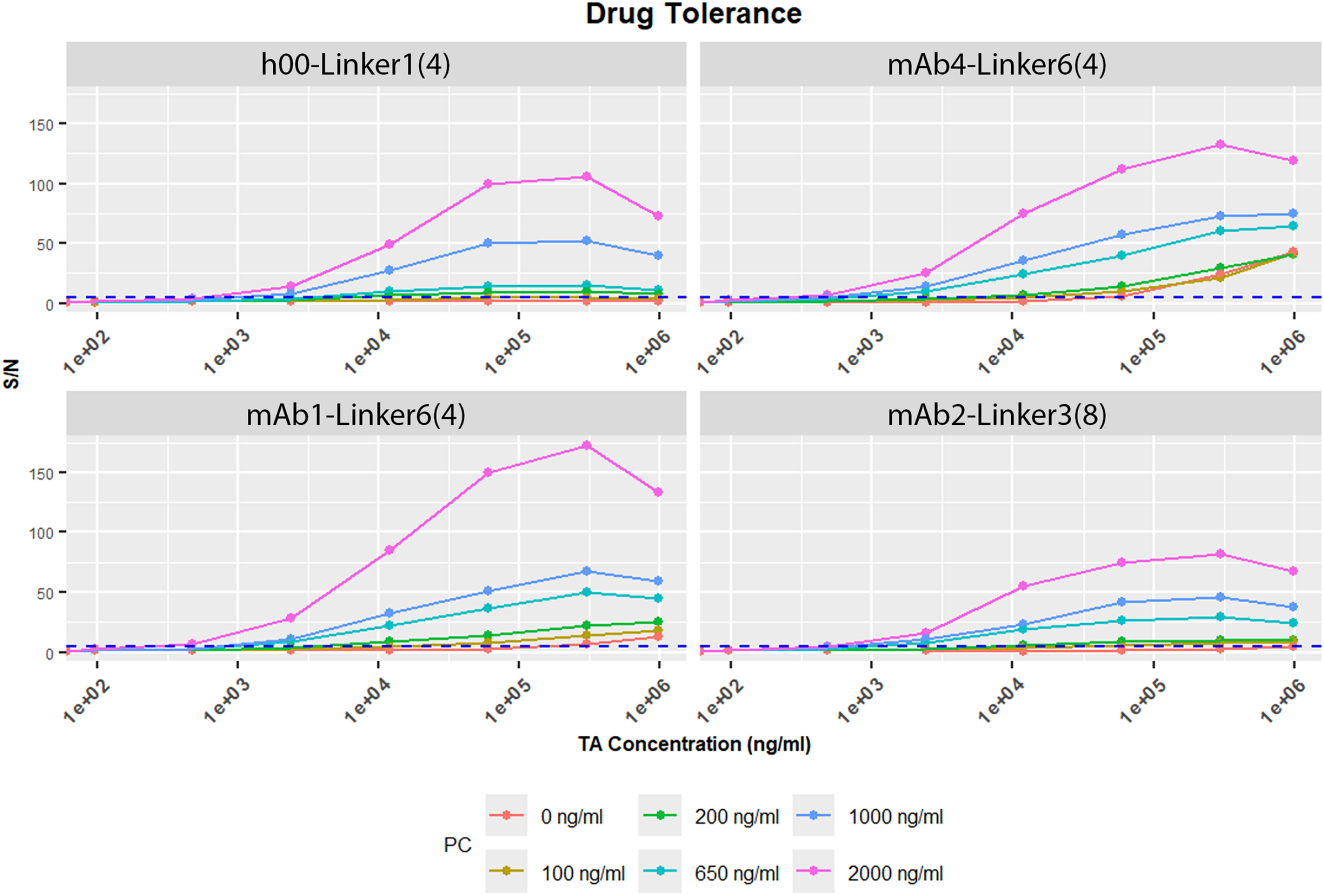
Drug tolerance data used to determine the amount of drug spikes. Drug tolerance was evaluated in four ADCs using various estimated cut points. Samples had PC levels from 2000 ng/mL to 100 ng/mL. The blue line indicates a S/N of 3 for estimating the cut point. All four ADCs were drug tolerate up to 1 mg/mL with 650 ng/ml of PC in sample.

The generic ADA assay adds drug to capture all free-formed ADA in an ADA/drug complex, which is then bound by an anti-human reagent and detected using an anti-species antibody (**Figure 1B**) compared to the gold standard bridging assay shown in **Figure 1A**. Instead of specific anti-idiotype (anti-ID) developed for each unique CDR, the surrogate positive antibody for the generic ADA assay is a species-specific mouse chimeric antibody towards the human light chain, shown in **Figure 1C**. The reagent used for cynomolgus is produced by replacing the IgG2a heavy chain and kappa light chain constant regions of a mouse anti-human kappa light chain antibody with cynomolgus IgG1 and lambda domains. The generic ADA assay not only provides an alternative method with universal reagents, but it also has a high drug tolerance built into the format. As shown in **Figure 2**, four ADCs with varying expected cut point ranges were tested for drug tolerance. Seven ADC concentrations ranging from 98 ng/ml to 1 mg/mL were added to lower PC levels at 100, 200, 650, 1000 and 2000 ng/mL in pooled cynomolgus plasma. After incubating for 1–2 hours, samples were diluted 1:100 in MRD with Rexxip HX and assay buffer, then detected using the gADA assay. After ADC was added, the S/N value rose in samples with ADC concentrations from 480 ng/mL to a maximum of 300000 ng/mL (4.8 ng/mL to 3000 ng/mL in-well concentration), then declined to the highest tested concentration of 1 mg/ml. All four ADCs showed this trend at PC levels above 650 ng/mL, with all S/N values for the highest 1 mg/mL drug concentration staying above 5, the higher estimate for a cut point. Utilizing this data, we determined the quantity of drug required for spiking into the complexation buffer to ensure complete capture of ADA present in the sample. This was achieved by dividing the maximum observed peak concentration by the MRD. In conclusion, this assay is drug tolerant up to 1 mg/mL at a low PC level at 650 ng/mL.

### 3.2 Cut point analysis of twenty-two distinct ADCs

**Figure 3:**
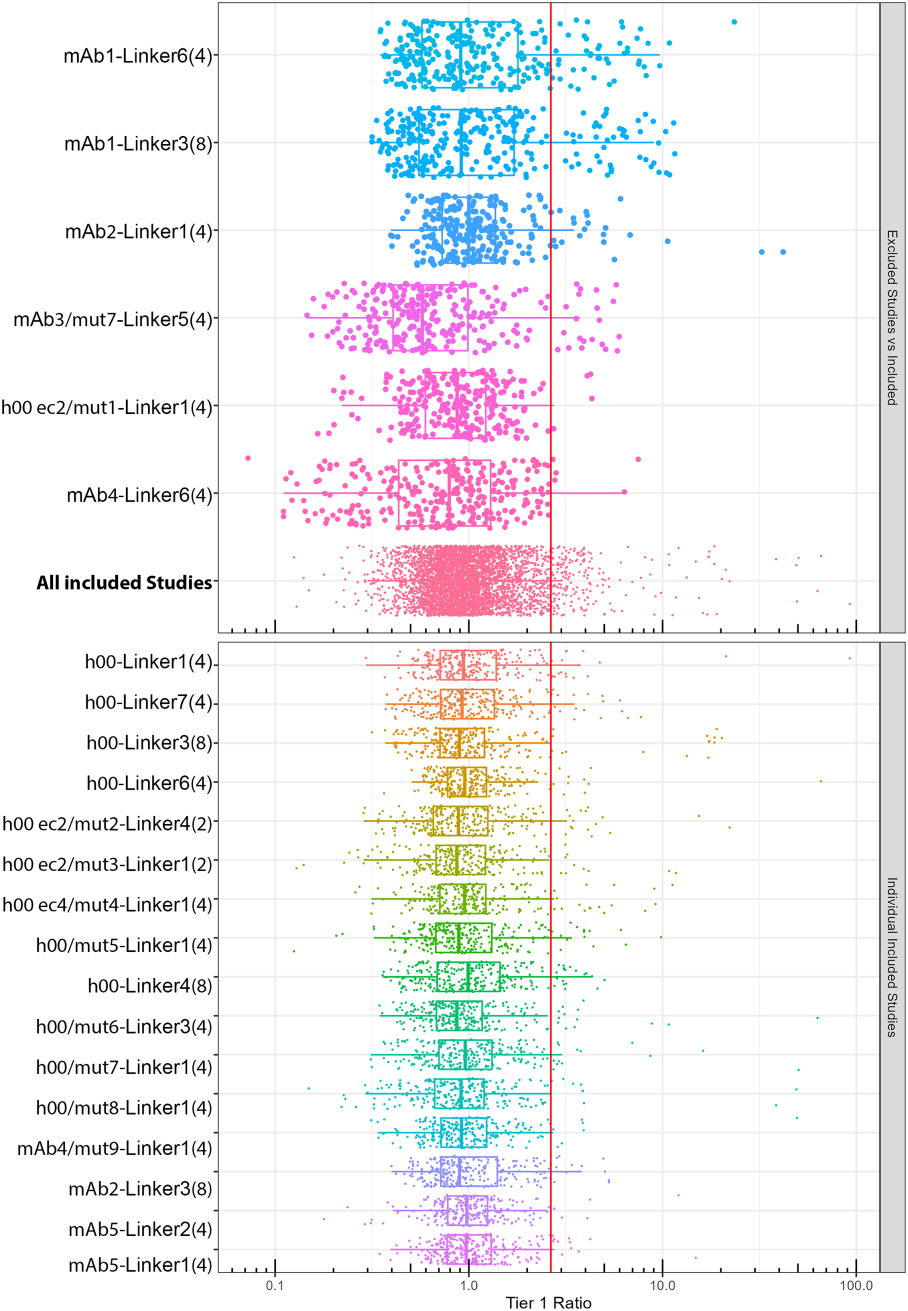
Cutpoint analysis: Plasma samples from fifty naïve cynomolguses were tested six times for each of twenty-two ADCs. The 95^th^ percentile cut points were calculated using a non-parametric method after removing outliers. A chi-squared test showed that 16 of 22 ADCs could share a common cut point of 2.64 (red line, lower panel). Data from these ADCs were pooled and compared with the excluded six ADCs, as shown in the upper panel.

Twenty-two ADCs with hIgG1k backbone were analyzed using the gADA method in plasma samples from 50 naïve cynomolgus subjects, each run six times over six days. Outliers for each ADC were removed using the 1.5 IQR method on log-transformed data. Demonstrated in **Table 1** and **Supplementary Figure 3**, the non-specific binding outliers were approximately 10% of all data collected. The cut point for each individual ADC was determined using a non-parametric approach to calculate the 95th percentile following outlier exclusion. As shown in **Table 1**, the cut points for each individual test article ranged from 1.88 to 6.79, false positive rates of after outlier exclusion averaged at 5.2%. Demonstrated in **Figure 3****, a** chi-squared test of homogeneity was deployed to determine similarity in cut points for all the ADCs. Sixteen out of twenty-two ADCs exhibited comparable data distributions, indicating the potential suitability for a universal cut point. Six out of twenty-two ADCs were excluded for exhibiting values that had statistically different distribution of data among the different naive plasma. Data from the remaining sixteen ADCs were consolidated, and a standardized cut point at the 95th percentile, as determined by quantile regression, was applied; this value was calculated to be 2.64.

### 3.3 Positive control setting for individual ADCs

**Figure 4:**
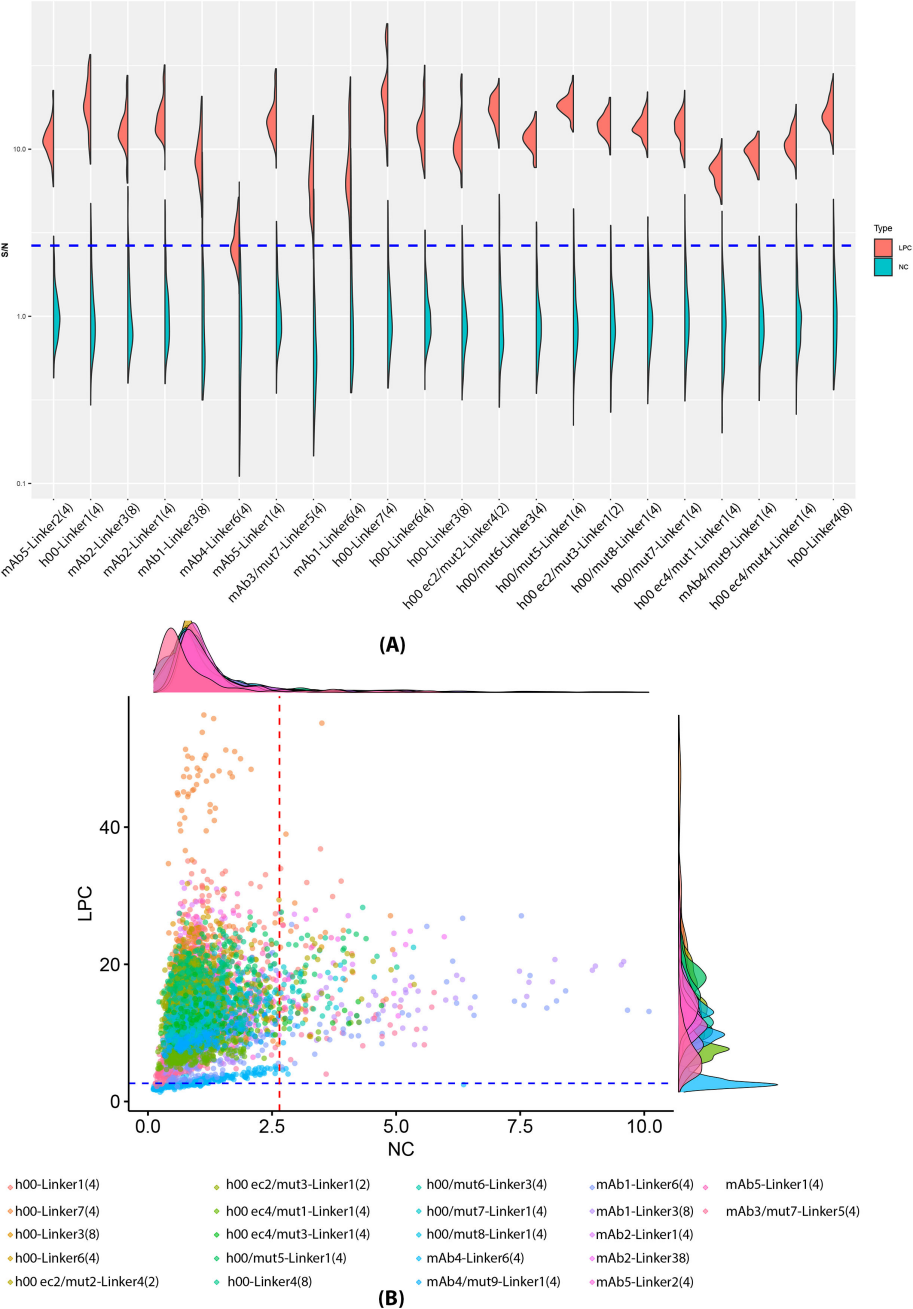
Naïve individual (NC) and LPC (650 ng/ml) spiked sample response in twenty-two unique ADCs A surrogate positive control targeting the light chain were spiked into the 50 cynomolgus at an LPC level of 650 ng/mL, with gADA ran with twenty-two ADCs. Naïve and LPC spiked individuals are compared shown in **(A, B)** Red and blue lines represent the 2.64 cut point.

**Figure 5:**
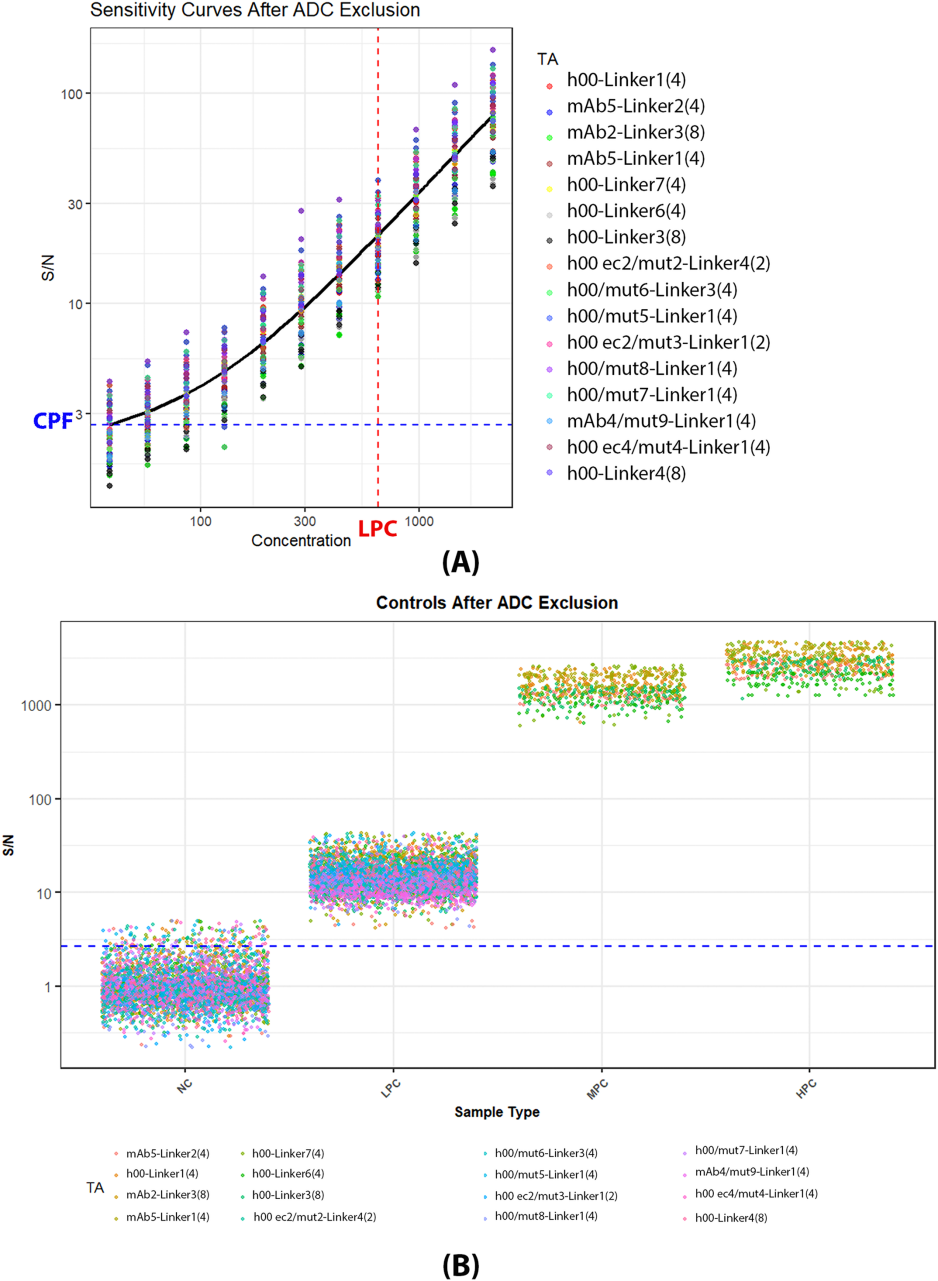
Combined sensitivity and positive control after homogeneity exclusion. Sensitivity curves for the 16 included ADCs were run. **(A)** show combined sensitivity curve with a 4-PL fit, blue line represents 2.64 universal cut point, red line presents the 650ng/mL LPC. MPC and HPC were set at 40,000 ng/mL and 100,000 ng/mL. **(B)** demonstrates the distribution of PCs compared to NC and cut point (blue line), all LPCs for 16 included ADCs passed the cut point, and within each individual ADC, HPC > MPC > LPC.

Baseline LPC were set at 650 ng/mL per drug tolerance data from **Figure 2**. Surrogate positive control was spiked in the same fifty cynomolgus subjects as the cut point screening. All samples were then tested with the gADA protocol across twenty-two ADC test articles. The outliers for LPCs were excluded using the 1.5 IQR method for each individual ADC. After outlier exclusion, all but one ADC had a false negative rate (FNR) above 5% under the application of 2.64 universal cut point. (**Supplementary Table 1****)** The failing ADC, mAb4-Linker6 (4), had a false negative rate of 50.17%. When compared to the cut point data, mAb4-Linker6 (4) was one of the six ADCs being excluded for the universal cut point analysis based on homogeneity. Demonstrated in **Figure 4A** and **Figure 4B**, mAb3/mut7-Linker5 (4), mAb1-Linker6 (4) and mAb1-Linker3 (8) have a significantly higher false positive rate for naïve samples, while mAb4-Linker6 (4) has higher false negative rates at 650ng/mL LPC level. When comparing the NC and LPC data distributions for each individual ADC, these four ADCs exhibit values where the right tail of NC is closest to the left tail of LPC. After excluding the six outlier ADCs, the LPC consistently falls above the cut point (**Figure 5B**). MPC and HPC were set at 40,000 ng/mL and 100,000 ng/mL, respectively, with each ADC showing HPC > MPC > LPC.

### 3.4 The average assay sensitivity for universal cut point ADCs remains below 100 ng/mL.

Four independent sensitivity curves serial diluted from 2193.8 to 38 ng/mL in pooled cynomolgus plasma were ran for all twenty-two ADCs, shown in **Supplementary Figure 2**. As listed in **Table 2**, at the universal cut point of 2.64, the average assay sensitivity of each individual ADC ranged from 7.17 to 455.3 ng/mL in all ADCs tested. After excluding the six outlier ADCs, assay sensitivity for each individual ADC ranged from 7.17 to 104.5 ng/mL with an upper limit of 89.3 to 185 ng/mL. Combined sensitivity of sixteen included ADC is demonstrated in both **Figure 5A** and **Table 2**, showed an average sensitivity of 51.2 ng/mL with an upper limit of 99.5 ng/mL close to the targeted value of 100ng/mL. Due to the differences in sensitivity among ADCs, a sensitivity assessment should be conducted for each ADC evaluated during study applications.

**Table 2:** Sensitivity of Individual ADC.

TA name	Sensitivity		
	Average	Upper limit	Lower limit
h00-Linker1 (4)	63.4	89.3	24.3
mAb5-Linker2 (4)	86.5	118.9	45.2
mAb2-Linker3 (8)	101.9	151.2	38.1
*mAb2-Linker1 (4)	78.3	147.3	NA
*mAb1-Linker3 (8)	128.3	195.7	58.4
*mAb4-Linker6 (4)	455.3	490.3	420.2
mAb5 -Linker1 (4)	71.1	150.7	NA
*mAb3/mut7-Linker5 (4)	168.1	278.6	71.2
*mAb1-Linker6 (4)	207.5	311.1	124.3
h00-Linker7 (4)	55.9	93.3	NA
h00-Linker6 (4)	91.18	156.2	NA
h00-Linker3 (8)	104.5	185.0	NA
h00 ec2/mut2 -Linker4 (2)	33.8	109.3	NA
h00/mut6-Linker3 (4)	63.5	123.9	NA
h00/mut5-Linker1 (4)	27.3	145.9	NA
h00 ec2/mut3-Linker1 (2)	28.9	139.0	NA
h00/mut8-Linker1 (4)	7.17	147.6	NA
h00/mut7-Linker1 (4)	23.1	113.3	NA
*h00 ec4/mut1-Linker1 (4)	67.5	101.4	NA
mAb4/mut9-Linker1 (4)	59.1	147.1	NA
h00/mut4-Linker1 (4)	35.9	108.3	NA
h00-Linker4 (8)	33.9	138.1	NA
22 TA Combined	62.7	118.6	NA
16 TA Combined	51.2	99.5	26.5

*ADCs are excluded from universal cut point analysis due to failed homogeneity test.

### 3.5 Canonical variable analysis reveals that cut point is mainly affected by mAb portion of the ADC

**Figure 6:**
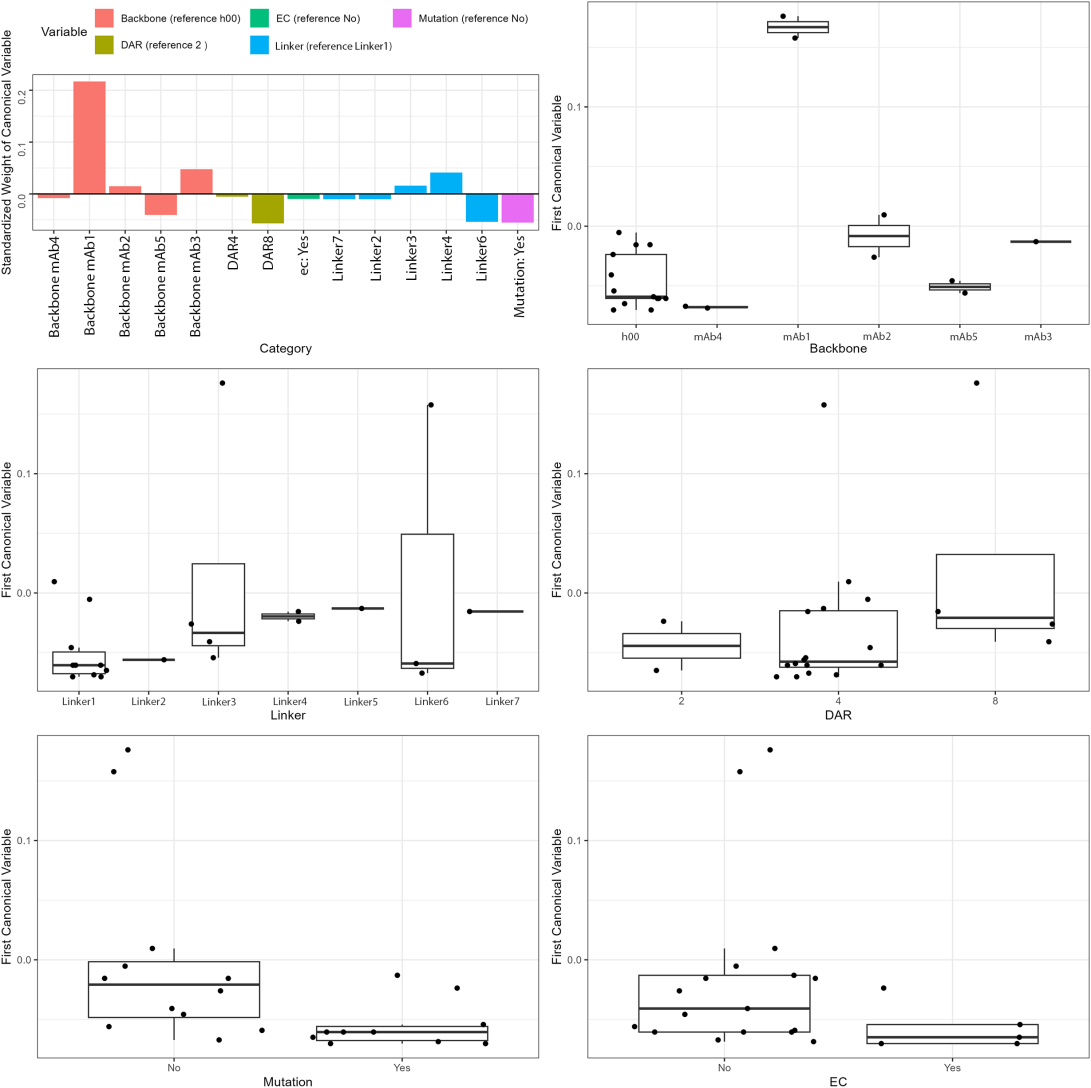
ADC component impact on cut point: a canonical correlation analysis. The effect of different ADC components, including antibody backbone, mutation, linker, DAR and conjugation method (EC), on gADA assay cut point were analyzed. Different antibody backbones have varied effect on cut point with one exception of mAb4 that significantly increased the cut point. No obvious trend is seen in different linkers and DAR due to variability. Presence of backbone mutations and ECs showed a negative trend for cut point.

An ADC is composed of linker, payloads and antibody backbone. Payload drug can be conjugated to an antibody backbone with different conjugation chemistry. Traditional conjugation approaches involve direct attachment to reactive cysteine residues on mAbs using agents such as maleimides, haloacetamides, or disulfide-reactive reagents. However, the classic conjugation method tends to result in a heterogeneous mixture of final conjugates with drug antibody ratio (DAR) ranging from 0 to 8 and an average DAR reported. To overcome variability in product, a homogeneous product can be achieved by site specific conjugation through various engineered cysteine (EC) approaches. Here, we compare the gADA assay performance of unique ADCs with different backbones/targets, backbone mutations, linkers, average DAR and conjugation methods using canonical variable analysis of the cut point. **Figure 6** shows that ADCs with different backbones have varying cut points compared to the non-targeting isotype control h00, with mAb1 displaying the highest background. When comparing the canonical values of linkers, there is no clear trend observed, likely due to high variability. Likewise, ADCs with EC conjugations do not demonstrate significant differences. Comparing ADCs with different DARs, DAR2 and DAR4 are comparable while DAR8 had higher canonical values. Certain FcRN mutations on the backbone also negatively affected the cut point, likely due to reduced binding of assay reagents. In summary, payload, DAR, and conjugation method minimally impact gADA assay, but performance still depends on the mAb backbone.

### 3.6 Proposed workflow to deploy the universal cut point for new study

**Figure 7:**
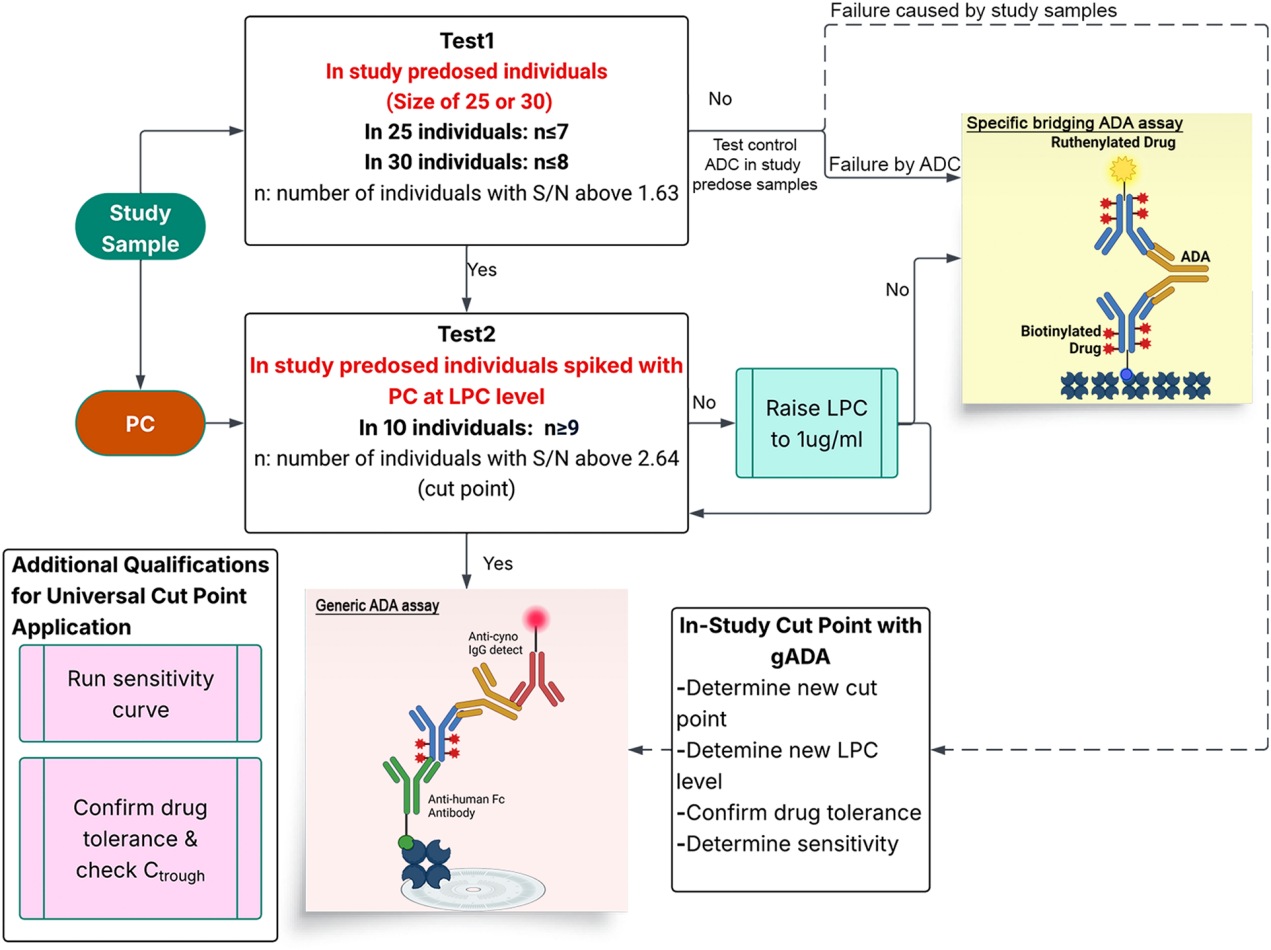
Flowchart for assay selection under GLP setting. Preassessments for new GLP studies will follow this workflow involving two-tiered testing. Test 1 evaluates 25 or 30 pre-dosed individuals: if less or equal to 7 out of 25, or 8 out of 30, exceeds the 85th percentile value of 1.63, the procedure is considered successful and advances to Test 2. If test 1 fails due to ADC incompatibility, a specific bridging assay will be carried out. If study individuals are the cause, then an in-study cut point with gADA assay can be considered. In Test 2, 10 pre-dosed individuals are spiked with PC at 650 ng/mL; if 9 out of 10 exceed the cut point value of 2.64, the test is deemed satisfactory. With further assessment of sensitivity and drug tolerance, the generic assay may then be implemented. If test 2 fails after LPC adjustment.

A workflow has been created using data from twenty-two ADCs to guide the use of the gADA assay for regulated studies, assess suitability for new test articles, and adjust assay parameters when necessary. Shown in **Figure 7**, assay suitability is assessed by a two-tiered approach using predosed in-study individual samples. We have established a boot strap method based on data distribution of the 16 ADCs included in cut point calculation. For the preliminary assessment, the 85^th^ percentile value of 1.63 was utilized to evaluate the suitability of an ADC. The universal 95^th^ percentile cut point of 2.64 will be deemed appropriate for naïve samples and progress to the subsequent test if less or equal to 8 out of 30 or 7 out of 25 pre-dosed samples, exceed the threshold of 1.63. Additionally, a surrogate positive antibody at a concentration of 650 ng/mL was added to ten randomly selected pre-dosed cynomolgus samples. If at least nine out of ten samples exceed the established cut point of 2.64, a second evaluation is initiated. Should more than 90% fall below the cut point, the LPC is increased to 1000 ng/mL and the assessment is repeated. Upon successful completion of the second evaluation, a general decision can be made for the deployment of gADA assay. Additional consideration and assessments including drug tolerance and sensitivity for unique TAs will also need to be taken into consideration. If the trough drug concentration (Ctrough) in the PK study is less than 0.7 mg/mL, no further analysis is required, allowing direct application of gADA parameters. If Ctrough exceeds 0.7 mg/mL, an additional drug tolerance assessment specific to that ADC will be performed. A sensitivity curve will be generated for each ADC evaluated, and the results will be reviewed as part of a purpose-specific assessment. If the baseline LPC of 650 ng/ml is 4-fold above sensitivity, LPC should be lowered and retested in the second test. In the case of a failed test 1, further investigation will be conducted to determine if it is caused by ADC or cynomolgus study samples through testing a controlled ADC in the pre-dosed samples. If ADC incompatibility is the cause, a dedicated specific ADA bridging assay will be deployed. If cynomolgus study samples are the reason, an in-study cut point approach with the gADA assay should be considered.

### 3.7 Case study demonstrating the application of the gADA assay for determining ADA domain specificity

**Figure 8:**
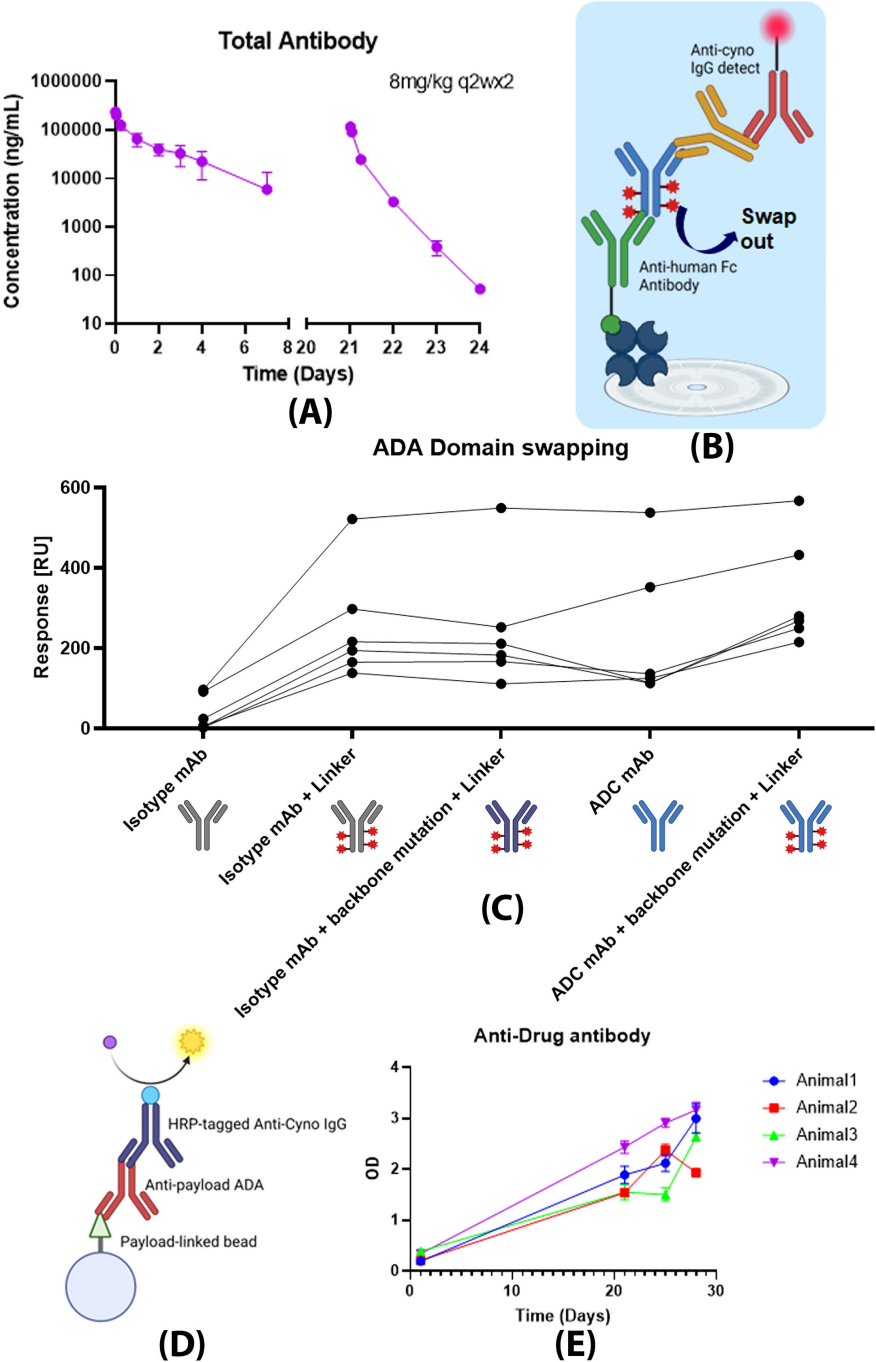
Case study utilizing gADA for domain specificity. **(A)** Presents total antibody concentrations measured in a cynomolgus pharmacokinetic (PK) study at an 8 mg/kg Q2W2 dosing regimen, with the second dose demonstrating evidence of accelerated clearance. Whole study design is described in **Supplementary Information**. ADA was analyzed for predose and trough-level samples. In the trough-level samples, alternative ADCs containing distinct protein therapeutic components were substituted for the ADC therapy depicted in **(B)**. **(C)** Assay responses for therapeutic agents (TAs) with different ADC domains were assessed across selected ADA-positive animals tested (n=6), with each animal depicted as a separate data point on the graph. The responses indicate anti-drug antibodies (ADA) directed toward both the backbone and linker regions. **(D)** A direct ELISA assay was performed to detect linker-specific ADA. The data shown in panel **(E)** corroborates the presence of ADA targeting the linker.

Though gADA is designed as a screening assay, additional assessment can be carried out for domain specificity. Here, we show a case study where we used the gADA assay to assess the specific domains ADA targeted towards. As illustrated in **Figure 8A**, rapid clearance was observed following the second administration of total antibody pharmacokinetics in a study involving cynomolgus monkeys receiving a therapeutic ADC at a dose of 8 mg/mL under a two dose every two-week (Q2W2) dosing regimen. ADA was detected from day 7 of the first dose throughout the second dose, S/N shown in **Supplementary table 2**. To determine the ADA targeting domain, ADCs containing individual components of the therapeutic ADC were used for complexation formation in the assay (**Figure 8B**). A nontargeting isotype control mAb was used as backbone control. The presence of the ADC linker on the backbone control substantially enhances the response, irrespective of whether it contains a LALA backbone mutation from the ADC therapeutic. When evaluating just the ADC backbone mAb, the response is also significantly higher compared to isotype control. (**Figure 8C**) The results indicate ADA towards both the backbone and the linker in the second dose. To confirm the finding, a direct bead ELISA (method described in **Supplementary Information**) was performed for ADA samples. In this assay, the linker payload was covalently attached to an agarose bead to capture cynomolgus ADA targeting the linker, as illustrated in **Figure 8D**. According to **Figure 8E**, samples from day 21 of the second dose showed an increased signal, which provides additional evidence for the presence of ADA specific to the linker.

## 4 Discussion

Over the past decade, the understanding of preclinical ADA assay benefits has evolved. While immunogenicity in animals does not predict human responses, assessing it remains valuable due to the expanding list of immune modulators in drug development. Using ADA to understand toxicology and drug exposure from preclinical studies can be crucial, though not always necessary. The challenge lies in balancing the need for immunogenicity assessment with resource constraints, since ADA assay development is time-consuming and costly, complicated further by limited availability of monkey serum/plasma. Currently a bridging assay that uses labelled drug as both capture and detection reagent is still the gold standard ADA assay format. Anti-ID antibodies serve as surrogate positive controls in the development of bridging ADA assays. Labelled anti-ID can also be applied as reagents for total antibody PK assays; however, their development incurs significant costs, especially in the preclinical phase. The increasing use of generic methods for monitoring total antibodies in cynomolgus pharmacokinetic studies raises important considerations regarding the cost-effectiveness of anti-ID development. Additionally, it prompts an evaluation of alternative approaches for detecting ADA in preclinical species that do not require anti-ID reagents. The bridging assay is limited by its sensitivity to preexisting drug, requiring labor-intensive, and potentially destructive, acid dissociation to extract bound ADA from the ADA/drug complex. The drug tolerance of a bridging assay is frequently limited, even when an extraction process is used. This limitation becomes apparent in TK studies, where animals receive high doses and substantial amounts of the drug are detected even at trough levels.

To develop an effective generic assay, we identified four key requirements: high drug tolerance for elevated concentrations in non-clinical studies; use of only generic reagents to avoid time-consuming development; sufficient signal-to-noise ratio for immunogenicity comparisons; and rapid implementation without the need to establish a separate cut point for each assay.

Several studies have proposed generic assays to replace specific ADA assays in nonclinical research. Carrasco-Triguero et al. (37) developed an assay meeting most criteria by spiking samples with excess drug (or ADC) for built-in drug tolerance, using anti-human Fc capture suited for IgG1/IgG4-based ADCs. While described as plug-and-play, this method lacked sufficient dynamic range for S/N evaluation, which is crucial for assessing ADA strength in PK studies. At WRIB 2023, we presented a Gyros-based assay offering enhanced dynamic range and drug tolerance. Li et al. (36) later published a similar format using four monoclonal antibodies and found that Gyrolab platform produced the best S/N compared to ELISA. Gyrolab CD’s bead column had high capacity, supporting near-saturating test article amounts for full drug tolerance—important in preclinical settings where dosing and antibody half-lives are high. We focused on evaluating an internal Gyrolab platform ADA assay utility in ADCs where linkers could have a significant effect on binding and matrix interference and provided a workflow guideline for assay use in regulated studies.

During background and cut point analysis, we observed that most ADCs showed similar backgrounds in the general assay, suggesting potential for a universal or generic cut point. This would simplify assay utility, since the drug serves as part of the reagent. To assess background variation with different antibodies, we tested 22 ADCs featuring various drug linkers, IgG1 backbones, backbone mutations, engineered cysteines, and variable regions compared to the non-binding h00 control antibody.

Using canonical component analysis, we evaluated whether any components caused differing backgrounds that might affect the cut point (**Figure 6**). While backbone modifications could impact positive control binding due to Fc region changes, most ADCs exhibited backgrounds within a similar range. Linker type, DAR, and mutations—including engineered cysteines—showed scattered but insignificant differences. The mAb1 variable region was the primary source of variance compared to h00, with certain Fc and ec mutations showing more negative spread without consistent effects across backbones. DAR did not significantly distort results, and neither steric hindrance nor lipophilicity contributed notable background variation.

Overall, most ADCs clustered around a single cut point, aside from a few outliers. We used a 95% cut point analysis due to limited data supporting a universal 99% cut point, this choice balances statistical robustness and practicality. Although a 99% cut point may be ideal, combining the 95% cut point with S/N provides sufficient information for exposure interpretation.

Any generic assay requires suitable positive control. This assay uses an anti-human Fc antibody engineered by swapping the IgG2a heavy and kappa light chain domains for cynomolgus macaque IgG1 and lambda domains, creating a chimera. This control produced strong binding across 21 of 22 ADCs tested (excluding mAb4-Linker6, for unclear reasons). Even antibodies with Fc mutations showed sufficient binding to be considered positive by cut point analysis, as shown in **Figure 4B**. The generic LPC met the criteria for all but one ADC and was effective at nonclinical assay sensitivity under 1000 ng/mL per industry guidance (17). While positive controls ensure assay reliability, their concentration can be reduced or reassessed initially, as strict FDA stringency may not always be required.

Before applying a generic assay and cut point to new ADCs, certain checks are necessary. **Figure 7** presents a flow chart for GLP studies with two steps: First, test serum samples from at least 25–30 predose animals (or naïve substitutes) with the new ADC as reagent. Calculate S/N against the control pool; if less or equal than 8 out of 30 samples or 7 out of 25 exceed 1.63, proceed to the sequential test. Otherwise, conduct further assessment to determine whether the failure is attributable to ADC or study samples. If incompatibility with ADC is identified as the cause, a specific bridging assay will be used; if not, an in-study cut point with the gADA assay can be considered. The sequential test involves spiking predose samples at LPC levels; if criteria are met, apply the generic assay with the 2.64 universal cut point, if not, it is unapplicable. Additional qualification experiments—such as adjusting LPC through a PC sensitivity curve or confirming drug tolerance—may be added as fit for purpose. In studies with a small sample size of less than 25 animals, where no statistical cut off can be drawn with first test, unless all the pre dosed animals have S/N under 1.63, an in-study cut point for will be recommended for gADA.

This work focuses on assessing the utilization of Gyrolab gADA assay in IgG1 ADC therapeutics through a universal cut point and purposing a workflow guideline. In the future, we plan to look at its performance in other IgG subclass and other antibody-conjugated modalities. We showed the utility of this assay for ADA domain characterization for ADCs in a non-regulated study where PK was affected by ADA (**Supplementary Information**). The study had a small sample size of 10 but all pre dosed individuals had S/N under 1.63. Due to the fit-for-purpose non-regulated use, we still applied the universal cut point after confirming LPC performance, assay sensitivity and drug tolerance. In context of regulated use, we cross compared results with a specific GLP bridging assay using historical GLP study samples, the gADA assay demonstrated a marginally increased ADA positive rate (data not shown). This observation is consistent with findings reported by comparable generic assays (36). However, due to the age of the specimen it is difficult to determine if it stems from the assay or due to sample discrepancy. It is still a continuing effort to compare generic assay results with various regulated bridging assays and ADA subtypes will also be investigated.

## 5 Conclusion

The generic ADA assay, using a standard positive control and universal cut point of 2.64, offers an efficient, adaptable solution for most ADCs in non-clinical studies. It saves time and costs by removing the need for separate assay development for each drug candidate.

## Data Availability

The raw data supporting the conclusions of this article will be made available by the authors, without undue reservation.
