# Supplementary material for: Development and implementation of a gyrolab-based generic anti-drug antibody assay for antibody-drug conjugates in cynomolgus monkey studies
Source: Front Immunol. 2025 Nov 18;16:1711816. doi: 10.3389/fimmu.2025.1711816 (PMC12670146; doi:10.3389/fimmu.2025.1711816)
Supplement: Supplementary file 1 [file DataSheet1.pdf]

Supplementary Information

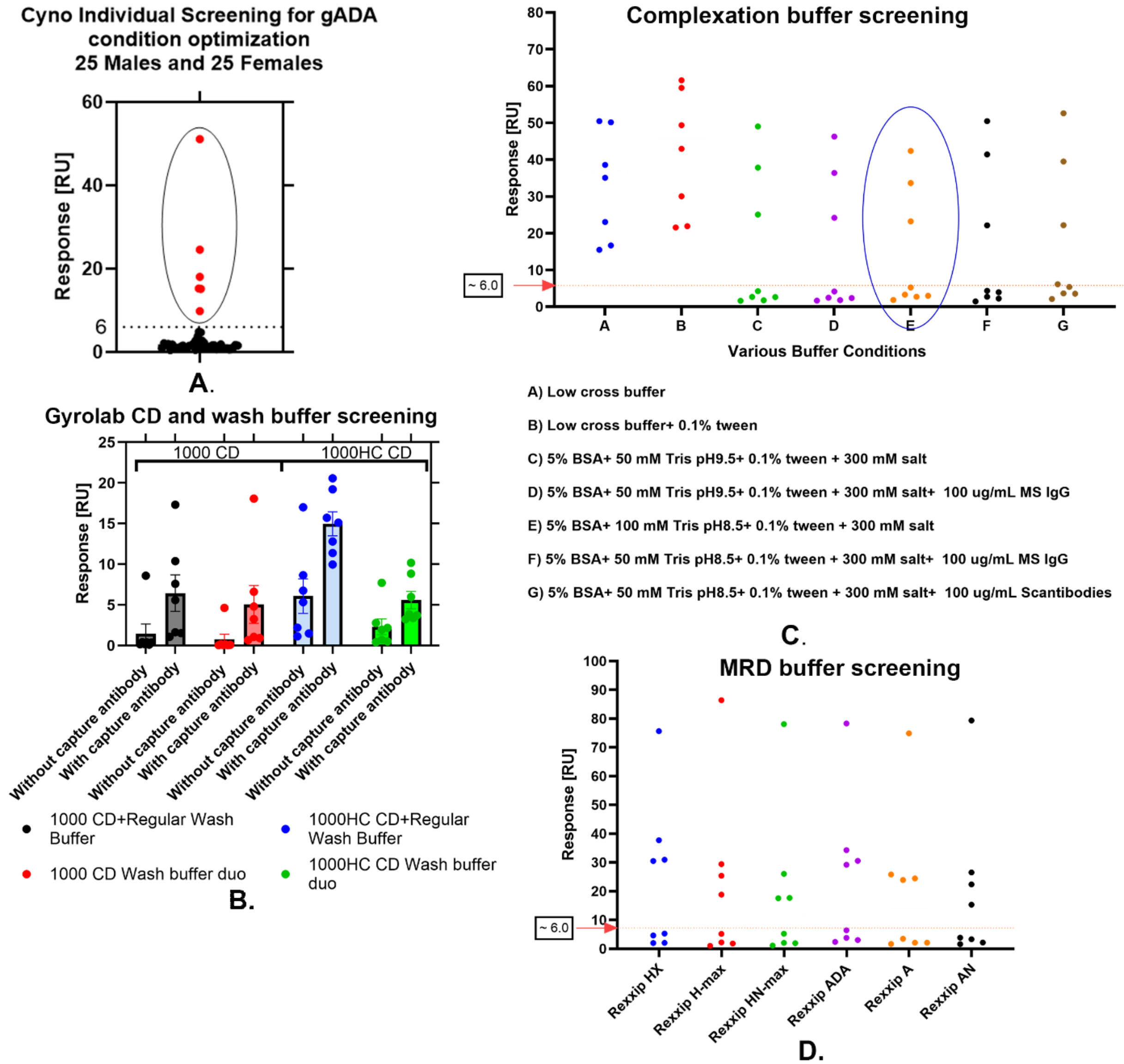

Supplementary Figure 1. Assay condition optimization with h00-Linker1(4)

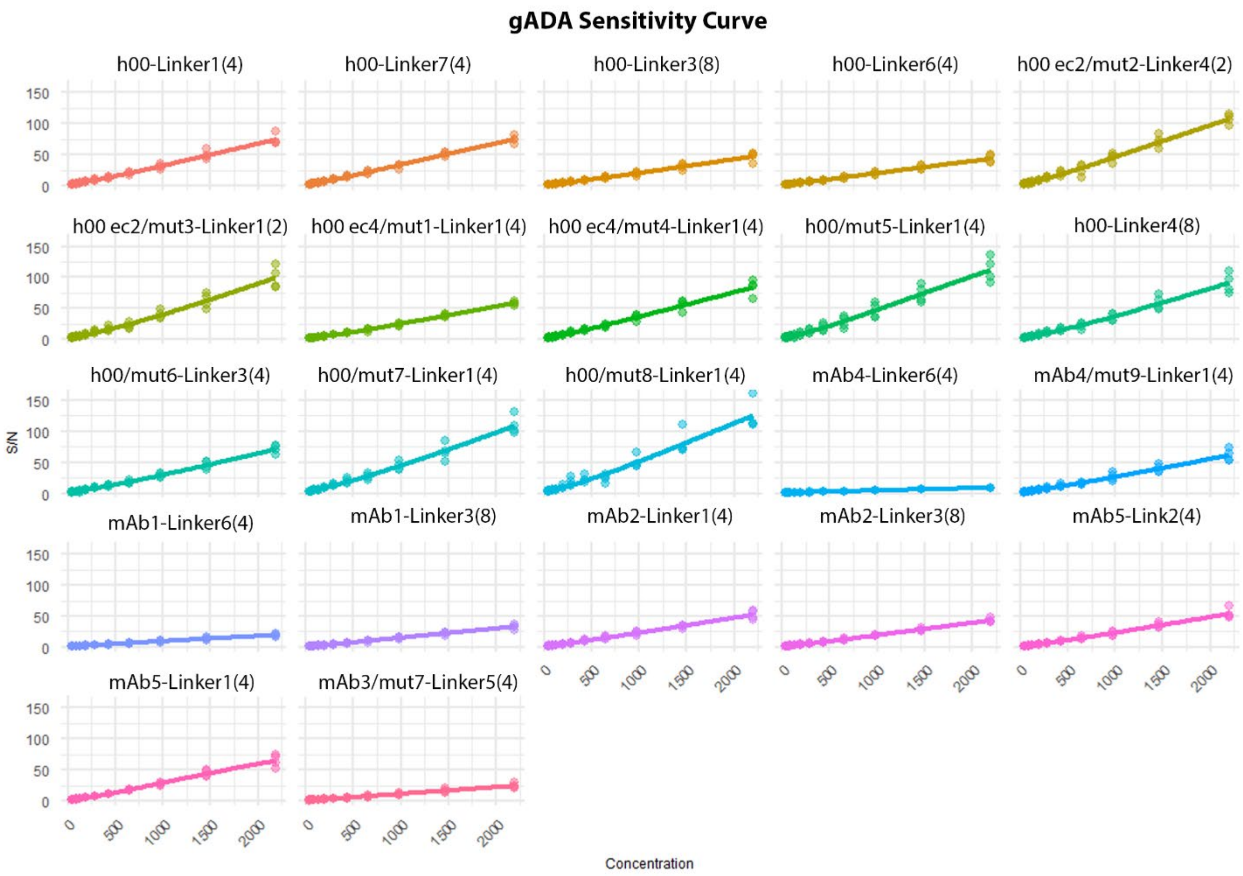

Supplementary Figure 2. Sensitivity curve of twenty-two ADCs

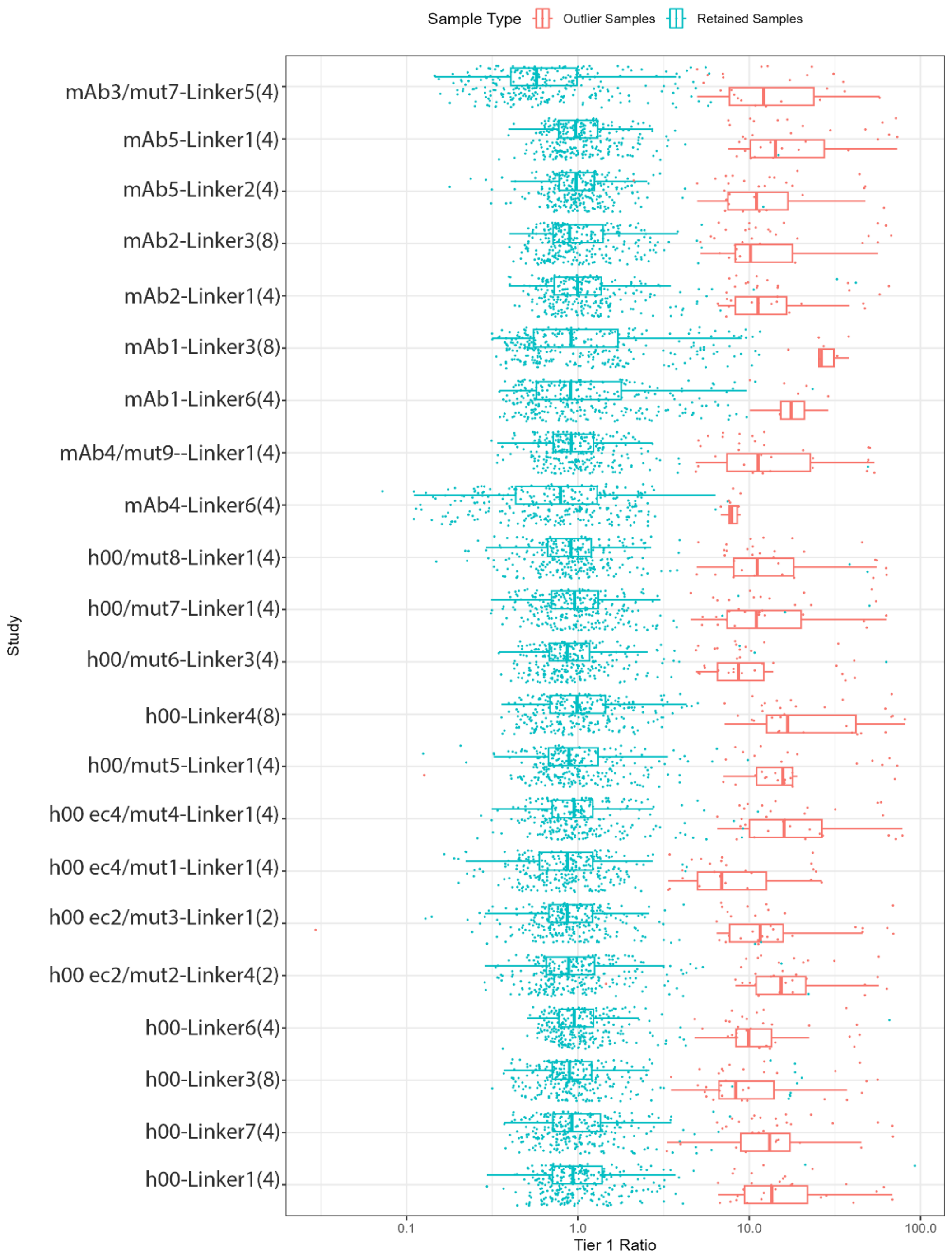

Supplementary Figure 3. Outlier exclusion for naïve samples from different ADCs

Supplementary Table 1. LPC false positive rate with universal cut point

TA	Count above Cut point all data	Count all data	%Percentage FNR for all data	Count above Cut point after outlier exclusion	Total data after outlier exclusion	%Percentage FNR after outlier exclusion
*mAb3/mut7-Linker5(4)	4	300	1.33%	3	291	1.03%
h00/mut5-Linker1(4)	2	300	0.67%	0	265	0.00%
h00/mut7-Linker1(4)	0	299	0.00%	0	272	0.00%
h00/mut8-Linker1(4)	0	300	0.00%	0	251	0.00%
h00 ec2/mut2-Linker4(2)	0	300	0.00%	0	269	0.00%
h00 ec2/mut3-Linker1(2)	2	300	0.67%	0	270	0.00%
*h00 ec4/mut1-Linker1(4)	0	300	0.00%	0	284	0.00%
h00 ec4/mut4-Linker1(4)	0	300	0.00%	0	273	0.00%
h00/mut6-Linker3(4)	0	300	0.00%	0	272	0.00%
h00-Linker4(8)	0	300	0.00%	0	280	0.00%
h00-Linker1(4)	0	300	0.00%	0	289	0.00%
h00-Linker7(4)	0	300	0.00%	0	284	0.00%
h00-Linker3(8)	0	300	0.00%	0	289	0.00%
h00-Linker6(4)	0	300	0.00%	0	289	0.00%
mAb4/mut9-Linker1(4)	0	300	0.00%	0	271	0.00%
*mAb4-Linker6(4)	146	300	48.67%	145	289	50.17%
*mAb1-Linker3(8)	0	300	0.00%	0	289	0.00%
*mAb1-Linker6(4)	1	300	0.33%	1	299	0.33%
mAb2-Linker3(8)	0	300	0.00%	0	278	0.00%
*mAb2-Linker1(4)	0	300	0.00%	0	289	0.00%
mAb5-Linker1(4)	0	300	0.00%	0	281	0.00%
mAb5-Linker2(4)	0	300	0.00%	0	258	0.00%

Test articles with * are excluded from the cut point analysis

Supplementary Table 2. ADA signal-to-noise data in Cynomolgus samples

Dose level (mg/kg)	Animal	Day 0 Predosed	Day7	Day21 Predosed	Day 28
8mg/kg	Animal1	0.9	*130.9	*284.93	*274.7
	Animal2	1.3	*127.0	*282.4	*239.6
	Animal3	0.7	*82.2	*285.7	*243.7
	Animal4	0.7	*148.6	*283.8	*246.1
4mg/kg	Animal5	1.1	*78.4	*180.7	*183.5
	Animal6	1.3	*97.2	*141.9	*180.3
	Animal7	1.1	*98.6	*186.8	*176.9

1mg/kg	Animal8	1.1	*25.2	*130.0	*183.6
	Animal9	1.2	*31.9	*160.2	*185.7
	Animal10	1.0	*125.4	*179.9	*176.9

*Samples are ADA positive confirmed with gADA assay cut point of 2.64

Supplementary Methods

Gyrolab gADA assay condition optimization

Due to the used of an anti-cynomolgus detection reagent, the matrix background is a concern and was optimized through optimizing buffer content, Gyrolab CDs and wash conditions. Gyrolab gADA assay condition optimized by first screening fifty cynomolgus individuals in baseline conditions where PTB (1% BSA, 0.1% Tween in PBS) were used as complexation buffer, Rexxip HX were used as MRD buffer, and 1000 CD were run with 1000-3W-04 methods. Raw responses were shown in **Supplementary Figure1A**, high response individuals were identified and used for condition optimization. 4 high responders and 4 low responders were screened with multiple complexation buffer conditions containing different salt content, pH and blocking reagents (**Supplementary Figure1B**), complexation assay buffer E (5% BSA, 0.1% Tween20, 0.3M NaCl solution, 0.1M Tris, pH8.5) had the best performance and was further used in assay qualification. Under the baseline condition, different Rexxip buffers were tested for MRD dilution, and the results showed comparable responses (**Supplementary Figure 1D**). Under optimized conditions, regular and high content Gyrolab CDs, 1000 CD and 1000 HC CDs as well as wash conditions were compared, shown in **Supplementary Figure 1C**. Conditions were looked at with and without capture reagent to determine nonspecific binding to the system. As expected, high content CDs resulted in higher background and are not applicable in this assay. Despite the non-cross reactivity of anti-human Fc capture antibody with cynomolgus IgG, some individuals still showed high background and has to be excluded as outliers.

Cynomolgus ADA domain specificity study design

Cynomolgus pharmacokinetics (PK) samples were obtained from a non-GLP study conducted in inotiv by legacy Seagen for investigative research purpose. 10 animals were IV dosed with ADC at 1mg/kg, 4mg/kg and 8mg/kg multi-dose (Q2W2), where second dose were given after a two-week interval. Plasma samples were collected on day 0 (predose), day 0 10mins, day 0 1hr, day 0 6hr, day 1, day 2, day 3, day 4, day 7, day 21 predose, day 21 10mins, day 21 1hr, day 21 6hr, day 22, day 23, day 24, day 25, day 28 for PK analysis. ADA was looked at on day 0 (predose), day 7, day 21 predose and day 28 for reporting and PK exclusion. Signal to noise was calculated through normalizing to the NC pool average on each Gyrolab CD and with universal cut point of 2.64 applied, data shown in **Supplementary Table 2**. PK samples from day 0 (predose), day 21(predose), day 25 and day 28 were used to characterize payload targeting ADA through bead-based ELISA. ADA positive samples day 21 predose samples from 8mg/kg dose group and 4mg/kg dose group were used to look at ADA domain specificity.

Bead-based ELISA to detect payload targeting ADA

300 µL of payload-covalently linked agarose beads (Cube Biotech #51103) were added to 4200 µL PBST following thorough vortexing. 150 µL of the bead-PBST mixture was dispensed into each

well of a 96-well V-bottom plate. The beads were washed by centrifuging the plate at 750 x g for 5 minutes, after which the supernatant was removed promptly. The wash was repeated 3 times. Beads were resuspended in 200 µL PTB buffer (5% BSA in PBST), sealed and shaken at 800 rpm at room temperature for 1hr. After blocking the beads were further washed with PBST 3 times and spun down. A mouse anti-payload antibody was used as surrogate control to confirm performance of the assay, which was further detected by an HRP tagged anti-mouse antibody. PCs were prepared by spiking the mouse anti-payload antibody at 1000, 5000, 25000 ng/mL into naïve pooled cyno plasma. Samples, PC and NC were diluted 1:20 in assay buffer (1% BSA in PBST), with 200 µL added to each well of beads. The plate was then shaken and incubated at room temperature for 1 hr. Beads were further washed 3 times with PBST. HRP tagged anti-mouse antibody was diluted 1:500 and HRP tagged anti-cyno antibody was diluted 1:4000 in assay buffer. 100 µL diluted anti-mouse detection antibody was added to the PC/bead wells and anti-cyno detection was added to the samples/bead wells, shaken and incubated for 1 hr. After 3-bead washes, 100 µL of prewarmed tetramethylbenzidine (TMB) substrate was added to the beads, shaken and incubated for another 30 mins. The reaction was stopped by adding 100 µL 1N HCl, with 100 µL supernatant transferred to a 96-well clear flat bottom plate. Absorbance was read at 450 nm with 630 nm as reference on the Clariostar plate reader.
